# Single-Cell RNA Sequencing of Peripheral Blood Reveals Immune Cell Signatures in Alzheimer’s Disease

**DOI:** 10.3389/fimmu.2021.645666

**Published:** 2021-08-09

**Authors:** Hui Xu, Jianping Jia

**Affiliations:** ^1^Innovation Center for Neurological Disorders and Department of Neurology, Xuanwu Hospital, Capital Medical University, National Clinical Research Center for Geriatric Diseases, Beijing, China; ^2^Beijing Key Laboratory of Geriatric Cognitive Disorders, Beijing, China; ^3^Clinical Center for Neurodegenerative Disease and Memory Impairment, Capital Medical University, Beijing, China; ^4^Center of Alzheimer’s Disease, Beijing Institute of Brain Disorders, Collaborative Innovation Center for Brain Disorders, Capital Medical University, Beijing, China; ^5^Key Laboratory of Neurodegenerative Diseases, Ministry of Education, Beijing, China

**Keywords:** Alzheimer’s disease, B cell receptor, single-cell RNA sequencing, T cell receptor, adaptive immunity

## Abstract

The peripheral immune system is thought to affect the pathology of the central nervous system in Alzheimer’s disease (AD). However, current knowledge is inadequate for understanding the characteristics of peripheral immune cells in AD. This study aimed to explore the molecular basis of peripheral immune cells and the features of adaptive immune repertoire at a single cell level. We profiled 36,849 peripheral blood mononuclear cells from AD patients with amyloid-positive status and normal controls with amyloid-negative status by 5’ single-cell transcriptome and immune repertoire sequencing using the cell ranger standard analysis procedure. We revealed five immune cell subsets: CD4+ T cells, CD8+ T cells, B cells, natural killer cells, and monocytes–macrophages cells, and disentangled the characteristic alterations of cell subset proportion and gene expression patterns in AD. Thirty-one cell type-specific key genes, comprising abundant human leukocyte antigen genes, and multiple immune-related pathways were identified by protein–protein interaction network and pathway enrichment analysis. We also found high-frequency amplification clonotypes in T and B cells and decreased diversity in T cells in AD. As clone amplification suggested the activation of an adaptive immune response against specific antigens, we speculated that the peripheral adaptive immune response, especially mediated by T cells, may have a role in the pathogenesis of AD. This finding may also contribute to further research regarding disease mechanism and the development of immune-related biomarkers or therapy.

## Introduction

Alzheimer’s disease (AD) is a progressive and eventually fatal neurodegenerative disease characterized by memory decline and disability. The hallmark of AD is the presence of plaques of the amyloid-β peptide (Aβ42) and neurofibrillary tangles (NFTs) of the phosphorylated protein tau. However, the classic hypothesis of the amyloid cascade cannot fully explain the pathogenesis of AD (1). Numerous genomics studies have suggested that immune function-related AD risk gene loci, such as triggering receptor expressed on myeloid cells 2(*TREM2*), cluster differentiation 33(*CD33*), and membrane spanning four domains subfamily A (*MS4A*), are closely related to the pathogenesis of AD (2, 3). The close functional relationship between the immune system and the central nervous system (CNS) is increasingly recognized. In the CNS, microglia, the most important immune cells in the nervous microenvironment, can convert to an activated state to phagocytize and remove amyloid beta (Aß), and they promote the release of inflammatory cytokines, thus accelerating neuronal damage (4). These phenomena suggest that dysregulation of the immune system is involved in the development of AD.

Moreover, the interplay of peripheral immune events and the CNS is also present in AD. Many types of immune cells, involving innate and adaptive immunity, containing monocytes, macrophages, neutrophils, and T cells from peripheral blood may participate in the pathogenesis of AD (5–8). Monocytes and macrophages express the CC chemokine receptor 2 and can be recruited to the inflammatory region of the brain through the CC motif chemokine ligand 2 or C–X3–C motif chemokine ligand 1 (9). By blocking the transport of neutrophils, ß 2 integrin lymphocyte function-associated antigen 1 leads to the depletion of neutrophils in mice with AD and improves cognitive impairment (10). T lymphocytes infiltrating into the brain through the choroid plexus participate in the adaptive immune response. CD8 T lymphocytes were detected in cerebrospinal fluid (CSF) of patients with AD (11). However, the current understanding of the distribution of peripheral blood immune cells in patients with AD is limited to flow cytometry study, and the cell type-specific functional status of immune cells, especially T and B cells, remains unclear. In addition, none of the studies has reported exploring the adaptive immune repertoire in the peripheral blood of patients with AD.

In order to explore the molecular basis of peripheral immune cells and the features of adaptive immune repertoire at a single cell level, we used 10X Genomics 5’ RNA, T cell receptor (TCR) and B cell receptor (BCR) sequencing technology of 36,849 peripheral blood mononuclear cells (PBMCs) from patients with AD and normal controls (NC), along with cell ranger standard analysis to define immune cell subsets, reveal the proportion of immune cell subsets and gene transcription states, and identify the key genes and pathways. Then we described the characteristics of the TCR and BCR repertoire from clonotypes, *V* and *J* genes skewed usage, amino acid length, and diversity of complementarity determining region 3 (CDR3) in patients with AD.

## Methods

### Participants

Three patients with AD older than 60 years of age, who were amyloid (18F-flutemetamol) positron emission tomography (PET)-positive, and two age-matched cognitively normal controls (NC), who were amyloid PET-negative, were included in the study (**Supplementary Table S1**). The clinical evaluation consisted of medical history, neurological examination, and a neuropsychological test battery. The inclusion and exclusion criteria for patients with AD and the NC are shown in the **Supplementary Methods**. All clinical investigations were conducted in accordance with the principles in the Declaration of Helsinki. The protocol of the study was approved by the Ethics Committees of Xuanwu Hospital. Prior to any study procedure, all participants of this study provided written informed consent.

### Peripheral Blood Mononuclear Cell Isolation and Cell Sorting

We collected the peripheral blood of all the participants at 7 a.m., and PBMCs were isolated by density-gradient centrifugation. After the PBMC suspension was resuspended in phosphate-buffered saline, followed by incubation at 4°C with CD45 antibodies for 20 min and 7-aminoactinomycin D for 10 min. MoFlo XDP (Beckman Coulter) was used for cell sorting. CD45+ cells were selected by gating on the basis of CD45 high, side-scatter low populations for subsequent single-cell RNA sequencing.

### Single-Cell 5’ Transcription and T Cell Receptor/B Cell Receptor Sequencing

The CD45+ cell suspension was stained with acridine orange/propidium iodide, and the number of cells was determined by the Countstar automatic cell-counting instrument (Countstar^®^ Rigel S6). Then the cell suspension was loaded in a 10× Chromium microfluidics system based on the manufacturer’s guidelines. Two sets of libraries were obtained from the 10× loaded samples: a 5’ gene expression messenger RNA library and a single-cell TCR and BCR library, using primers for TCR and BCR amplification as per the manufacturer’s instructions. Libraries were pooled together and run on separate lanes of a 150 base-paired, paired-end, flow cell using the Illumina NovaSeq 6000.

### Cell Ranger Pipeline and Cell Clusters Analysis

The Cell Ranger software (10X Genomics) obtained from https://support.10xgenomics.com/single-cell-gene-expression/software/downloads/ latest was used to perform barcode counting and unique molecular identifier counting after filtering and alignment to the GRCh37 (hg19) reference genome to generate the feature-barcode matrix and determine clusters. Dimensionality reduction was performed using principal component analysis, and the first ten principal components were used to generate clusters by the K-means algorithm and graph-based algorithm, respectively. Data analysis was performed through the Loupe Cell Browser software (10X Genomics) on Cloupe files displaying t-distributed stochastic neighbor embedding (t-SNE) projections of cell transcriptome.

### Functional and Pathway Enrichment Analysis

Next, the differential expression genes for each cluster were imported into Metascape (http://metascape.org/) for gene ontology analysis of biological processes and the Kyoto Encyclopedia of Genes and Genomes (KEGG), and reactome pathway analysis was performed with a false discovery rate (FDR) <0.01 as the cut-off value. Furthermore, similarity analysis was performed using the enriched terms based on the kappa value. If the kappa value between two terms was <0.3, the two terms were connected by edges and visualized by Cytoscape version 3.6.0 software.

### Protein–Protein Interaction Network Analysis

Protein–protein interaction network analysis (PPI) was performed using Metascape. The molecular complex detection (MCODE) algorithm was used to identify the closely related modules in the network.

### T Cell Receptor and B Cell Receptor Repertoire Analysis

We used the Loupe V(D)J browser to analyze the TCR and BCR clonotypes, and the *V* and *J* genes. After exporting the clonotype-related data that underwent standardized process analysis from the Loupe V(D)J software, we used the Excel script for analysis as follows: 1) the top 10 clonotype frequencies of T and B cells between the AD group and NC were compared, and the proportion of different clonotypes in each frequency interval of the AD group and NC was calculated. 2) The common clonotypes of T and B cells in the AD group and NC were described and visualized with the Venn diagram. 3) The diversities of the TCR and BCR CDR3 regions were evaluated by the InvSimpson index and Shannon–Weiner index, respectively. 4) We calculated and compared the amino acid sequence length of the CDR regions in the TCR and BCR between the AD group and NC. 5) The *V* and *J* gene usage combination reflects the diversity of clonotypes. The frequencies of *V* and *J* genes in each sample were calculated respectively, and the same *V* and *J* genes of the TCR and BCR in the AD group and NC were compared.

### Statistical Analyses

The chi-square test and Fisher’s exact test were used to compare the proportions between the groups. All statistical data were analyzed using SPSS 23.0 software (IBM Corp.).

## Results

### Single-Cell Transcriptome Profiling of Peripheral Blood Mononuclear Cells in Alzheimer’s Disease

**Figure 1:**
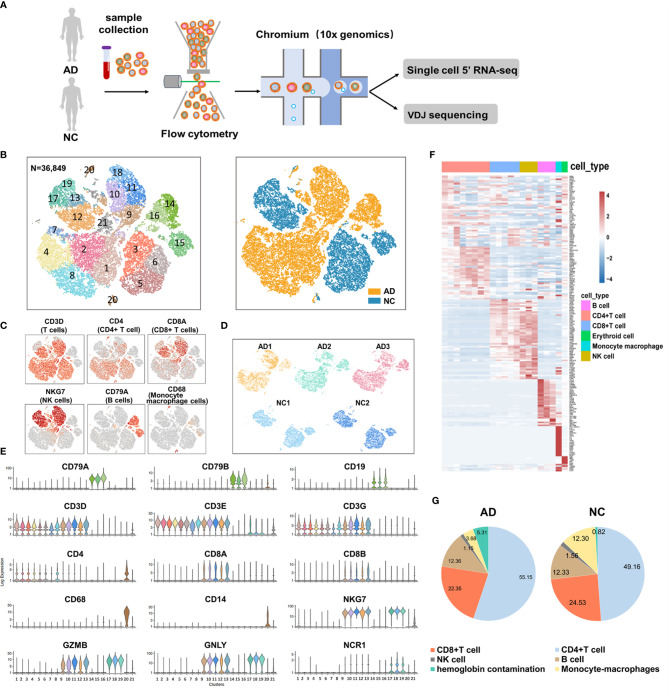
Single-cell gene expression analysis of peripheral blood mononuclear cells (PBMC) in Alzheimer’s disease (AD). **(A)** Experimental workflow for single-cell RNA analysis in the present study. **(B)** tSNE projection of 36,849 cells from PBMCs in the AD group and normal controls (NC). A total of five cell types were shown in **(B)**, including CD4+ T cells (clusters 1, 2, 3, 4, 5, 6, 7 and 8), CD8+ T cells (clusters 9, 10, 11, 12, and 13); B cells (clusters 14, 15 and 16); Natural killer (NK) cells (clusters 17, 18 and 19) and monocyte-macrophages cells (clusters 20). **(C, E)** Canonical cell surface markers define CD4+ T cells, CD8+ T cells, natural killer (NK) cells, B cells, and monocyte-macrophage cells. **(D)** tSNE projections of cell transcriptome in each sample in this study. **(F)** Heatmap of the top 20 marker genes from each cluster. **(G)** Proportion of the five types of immune cells in the AD group and NC.

We used fresh PBMCs derived from patients with AD and two NCs according to the inclusion and exclusion criteria. After single-cell 5’ gene expression sequencing and aggregating all sample data from Cell Ranger, we finally obtained 36,849 recovered cells, comprising 22,775 cells for patients with AD and 14,074 cells for NC (**Figure 1A**). Detailed information on data quality control is shown in **Supplementary Table S2** and **Supplementary Figure S1**. Using t-SNE analysis to visualize the cells in two-dimensional space, we found 21 clusters representing different cell types (**Figure 1B**). Five major immune cells types were identified, including CD4+ T cells (clusters 1, 2, 3, 4, 5, 6, 7, and 8) with canonical marker genes *CD3D*, *CD3E*, *CD3G*, and *CD4*; CD8+ T cells (clusters 9, 10, 11, 12, and 13) with marker genes *CD3D*, *CD3E*, *CD3G*, *CD8A*, and *CD8B*; B cells (clusters 14, 15, and 16) with marker genes *CD19*, *CD79A*, and *CD79B*; natural killer (NK) cells (clusters 17, 18, and 19) with marker genes *NKG7*, *GZMB*, *GNLY*, and *NCR1*; and monocyte–macrophage cells (cluster 20) with marker genes *CD14* and *CD68* (**Figures 1C, E**). In addition, cluster 21 highly expressed *HBB*, *HBA2*, *PF4*, and *PPBP* genes. Considering that cluster 21 may be mixed with hemoglobin and platelets in sample preparation, this cluster was excluded from the subsequent analysis. The top 20 featured genes of each cluster were displayed in the heat map (**Figure 1F** and **Supplementary Data 1**). We also found that AD and NC were separated in the t-SNE plot, suggesting that a major contrast existed in the gene expression patterns. Each sample had good consistency within the AD group or NC (**Figure 1D**). Furthermore, we compared the proportion of the five types of immune cells between the AD group and NC (**Figure 1G**). Numbers of CD4+ T cells were significantly increased in the AD group (55.15%) compared with NC (49.16%, P <0.01). In contrast, the proportion of CD8, NK, and monocyte–macrophage cells were significantly lower in the AD group than in NC (all P <0.01). The proportion of B cells was not significantly difference between the two groups.

### Functional and Pathway Enrichment Analysis of Distinct Immune Cells

**Figure 2:**
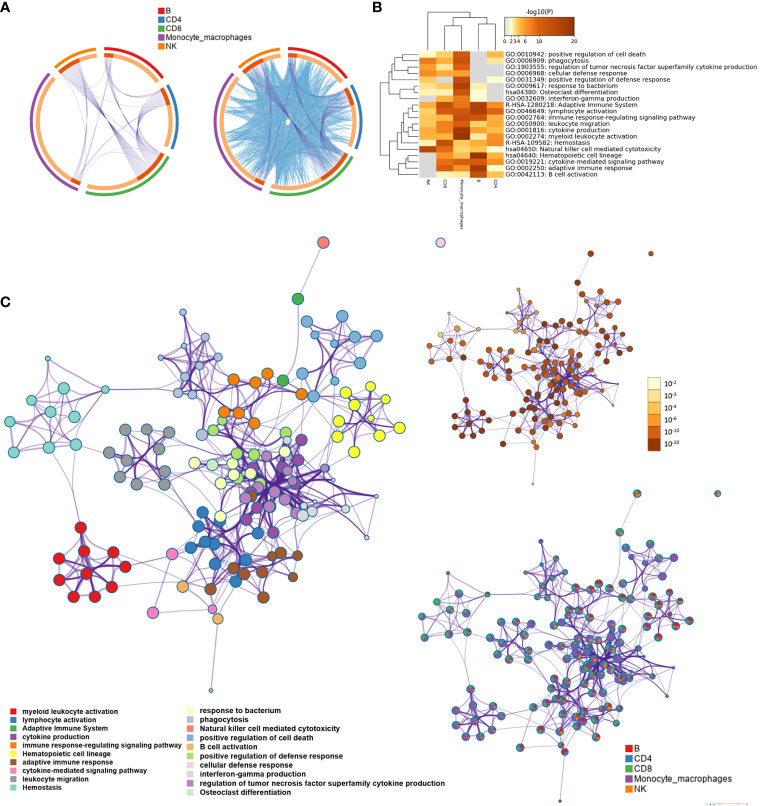
Functional and pathway enrichment analysis for B cell, CD4+ T cell, CD8+ T cell, natural killer (NK) cell, and monocyte-macrophage cell subsets. **(A)** The Circos plot shows how differentially expressed genes from the given immune cell subsets overlap. Each arc represents the identity of each gene list. Purple lines link the same gene that is shared by multiple gene lists. Blue lines link the different genes where they fall into the same ontology term (the term has to be statistically significantly enriched with a size no larger than 100). **(B)** The heatmap cells are colored based on the P-values of the enriched terms, and white cells indicate the lack of enrichment for that term. **(C)** Network of enriched terms; the nodes denote the enriched terms, and terms are connected by edges with a kappa statistic of >0.3. Enriched terms in the same cluster are denoted by the same color. In addition, the same enrichment network has its nodes colored based on the P-value and displayed as pies; different color sectors represent different subsets involved in the same enrichment pathway.

We screened the differentially expressed genes for each type of immune cell between the AD group and NC with | log2fc | >0.5 and FDR <0.05, respectively. The numbers of differentially expressed genes were 81 for B cells, 80 for CD4+ T cells, 117 for CD8+ T cells, 55 for NK cells, and 169 for monocyte–macrophage cells (**Supplementary Data 2**). Metascape was used to analyze multiple differentially expressed gene lists on three aspects of biological processes, KEGG, and the reactome pathway, respectively (**Supplementary Figure S2** and **Supplementary Data 3**). The Circos plot showed how genes from multiple gene lists overlap from five immune cell subsets, and the top 20 significantly enriched terms are shown in **Figures 2A, B**. Among the 20 enriched terms, eight pathways were shared by five types of immune cells, including “adaptive immune system”, “lymphocyte activation”, “immune response-regulating signal pathway”, “leukocyte migration”, “cytokine production,” “myeloid leukocyte activation”, “hemostasis”, and “natural killer cell-mediated cytotoxicity”. To further capture the relationships between the enriched terms, a subset of enriched terms was selected and rendered as a similarity network plot (**Figure 2C**).

### Protein–Protein Interaction Network and Key Gene Analysis

**Figure 3:**
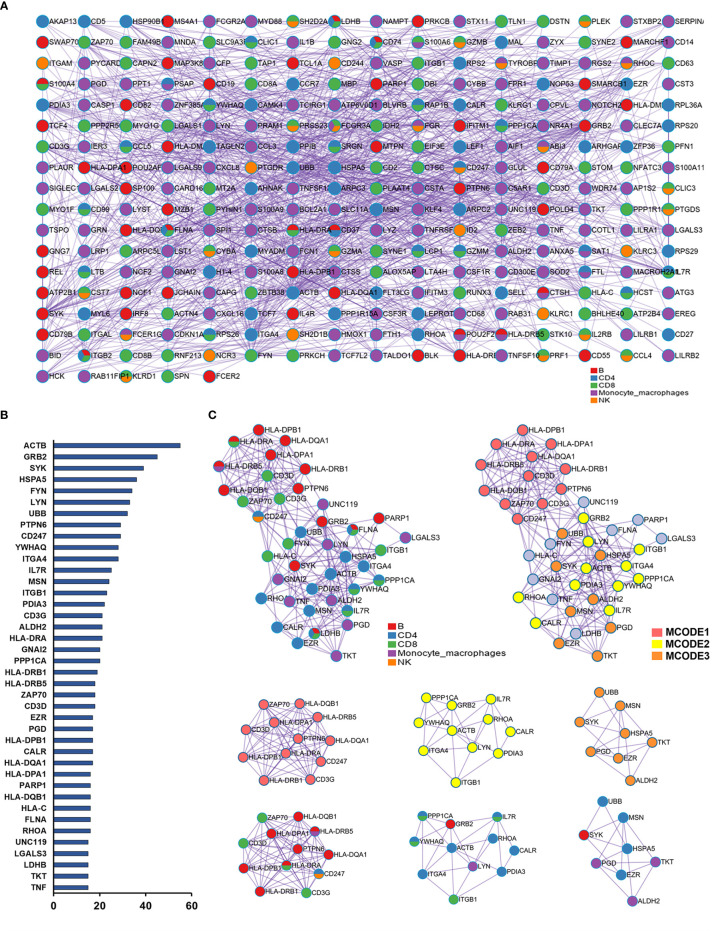
Protein–protein interaction (PPI) networks analysis. **(A)** PPI network of the differential expression genes between the Alzheimer’s disease (AD) group and normal controls (NC) from CD4+ T cell, CD8+ T cell, natural killer (NK) cell, B cell, and monocyte–macrophage cell subsets. The nodes are colored based on different subsets. **(B)** The top 40 genes identified though degree values in the PPI network. **(C)** The PPI subnetwork is constructed from the top 40 genes; three modules were identified by using the molecular complex detection (MCODE) algorithm, which comprised 31 key genes.

We integrated differentially expressed genes of different immune cell clusters. After removing the overlapped genes, 436 genes were obtained. To delineate the interactions among these genes, we constructed a PPI network using Metascape (**Figure 3A**). Moreover, we selected the top 40 genes based on the degree ranking method for sub-network analysis, and three modules were identified to be significant with the MCODE algorithm (**Figures 3B, C**). The three modules contained 31 genes, which were identified as the key genes (**Figure 3C**). The key genes in the MCODE1 module were *CD247*, *CD3D*, *CD3G*, *HLA-DPA1*, *HLA-DPB1*, *HLA-DQA1*, *HLA-DQB1*, *HLA-DRA*, *HLA-DRB1*, *HLA-DRB5*, *PTPN6*, and *ZAP70*. The T cell surface marker genes in this module were derived from CD8+ T cells, and abundant major histocompatibility complex (MHC) genes were derived from B cells. Key genes in the MCODE2 module were *ACTB*, *CALL*, *GRB2*, *IL7R*, *ITGA4*, *ITGB1*, *LYN*, *PDIA3*, *PPP1CA*, *RHOA*, and *YWAQ*. Most of the genes in the MCODE2 module were derived from CD4+ and CD8+ T cells. The key genes of the MCODE3 module were *ALDH2*, *EZR*, *HSPA5*, *MSN*, *PGD*, *SYK*, *TKT*, and *UBB*. These genes were mainly derived from CD4+ T cells and monocyte-macrophage cells. Finally, we performed function and pathway enrichment analysis for key genes in each module (**Supplementary Table S3**).

### T Cell Receptor Repertoire Analysis

**Figure 4:**
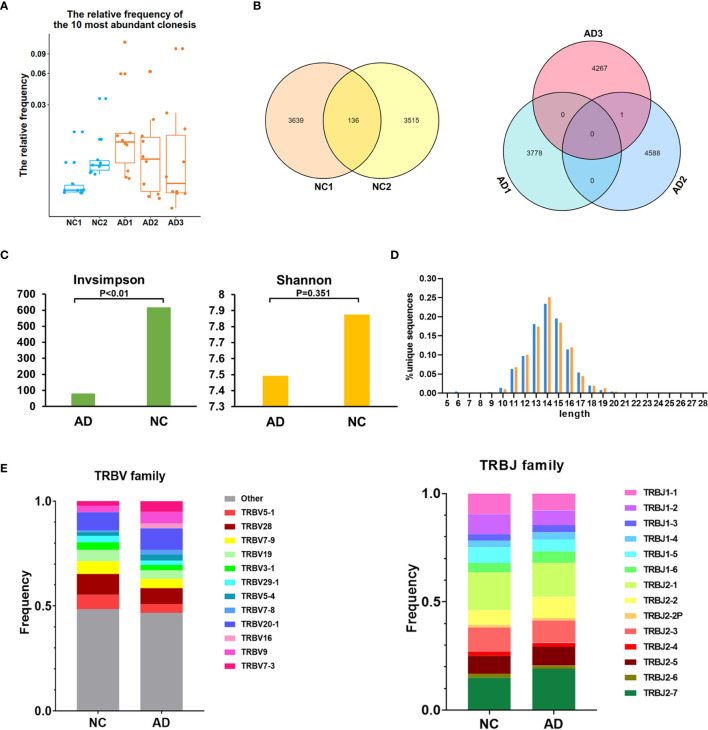
T cell receptor (TCR) repertoire analysis. **(A)** Box plot showing the top 10 high-frequency T cell clonotypes for each sample in this study. **(B)** Venn diagrams of the public T cell clonotypes within the Alzheimer’s disease (AD) group and normal controls (NC). **(C)** Averages of the InvSimpson index and Shannon–Weiner index of each sample are used to compare the TCR diversity of the AD group and NC. **(D)** Distribution of the CDR3 amino acid length of the TCR in the AD group and NC. **(E)** *V* and *J* genes usage frequency stacked histogram showing the distribution of common *V* and *J* genes of the TCR in the AD group and NC respectively.

The TCR repertoire of peripheral blood in the AD group and NC was analyzed by 10X Genomics single-cell V(D)J sequencing technology. The top 10 and top 100 clonotypes were compared between the AD group and NC. The frequency of the top 10 clonotypes was significantly higher in the AD group (18.8%, 3,178) than in NC (7.7%, 705; P <0.01). The frequency of the top 100 clonotypes was significantly increased in the AD group (25.7%, 4,350) compared with NC (19.8%, 1,821; P <0.01). A box plot of the top 10 high-frequency clonotypes for each sample is shown in **Figure 4A**. We analyzed the public clonotypes within the AD group and NC; 136 (1.9%) public clonotypes were found in NC, but no public T cell clonotypes were found in the AD group (**Figure 4B**).InvSimpson and Shannon–Weiner indexes were used to assess the diversity of the amino acid sequence in the CDR3 region. The InvSimpson were significantly decreased in the AD group compared with NC (80.66 versus [*vs.*] 617.65, P <0.01); however, this finding was not statistically significant for the Shannon–Weiner index (AD 7.49 *vs.* NC 7.87, P = 0.351) (**Figure 4C**). The reduction of diversity might have been caused by the oligoclonal expansion of TCR. In the analysis of the CDR3 amino acid length, we found that the most common amino acid lengths were 13, 14, and 15. A comparison of the frequency of different amino acid lengths between the AD group and NC revealed a statistically significant difference in only lengths 6, 10, 14, 15, 17, and 19 (**Figure 4D**).

To compare the frequency of the *V* and *J* gene of the TCR in the AD group and NC, a usage frequency stacked histogram was generated according to common usage frequency of the *V* and *J* gene (**Figure 4E**). *V* genes, including *TRBV5-1*, *TRBV7-3*, *TRBV9*, *TRBV16*, *TRBV20-1*, *TRBV7-8*, *TRBV5-4*, and *J* genes, including *TRBV5-1*, *TRBV7-3*, *TRBV9*, *TRBV16*, *TRBV20-1*, *TRBV7-8*, and *TRBV5-4*, showed a higher frequency in the AD group than in NC (P <0.05).The results indicating that the change of *V* and *J* genes usage spectrum in AD might be stimulated by some similar antigens.

### B Cell Receptor Repertoire Analysis

**Figure 5:**
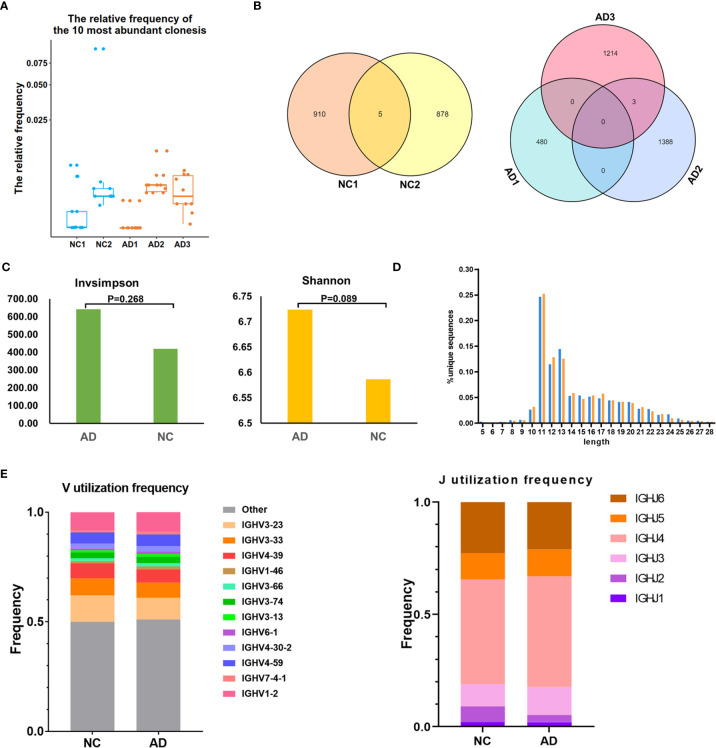
B cell receptor (BCR) repertoire analysis. **(A)** Box plot showing the top 10 high-frequency B cell clonotypes for each sample in the present study. **(B)** Venn diagrams of the public B cell clonotypes within the Alzheimer’s disease (AD) and normal controls (NC). **(C)** Averages of the InvSimpson index and Shannon–Weiner index of each sample are used to compare the BCR diversity of the AD group and NC. **(D)** Distribution of the CDR3 amino acid length of the BCR in the AD group and NC. **(E)** *V* and *J* genes usage frequency stacked histogram showing the distribution of common *V* and *J* genes of the BCR in the AD group and NC respectively.

For BCR clonotype analysis, both the top 10 and top 100 clonotypes were contrasted between the AD group and NC. The frequency of the top 10 clonotypes was higher in the AD group (7.7%, 256) than in NC (5.8%, 142; P <0.01), and the frequency of the top 100 clonotypes was higher in the AD group (26.0%, 865) than in NC (17.4%, 430; P <0.01). The box plot illustrates the top 10 high-frequency clonotypes for each sample (**Figure 5A**). The public clonotypes in both the AD group and NC were analyzed; five (0.2%) public clonotypes were found in NC, whereas no public T cell clonotypes were observed in the AD group (**Figure 5B**).

We further assessed the diversity of the amino acid sequence in the CDR3 region using the InvSimpson and Shannon–Weiner indexes. No statistically significant difference was found in any of the diversity indexes between the AD group and NC (InvSimpson index: 642.28 and NC 419.9, respectively; P = 0.268; Shannon–Weiner index: 6.72 and NC 6.59, respectively; P = 0.089) (**Figure 5C**). During the process of analyzing the CDR3 amino acid length, we found that the most common amino acid lengths were 11, 12, and 13. According to the comparison of the frequency of different lengths between the AD group and NC, statistically significant differences were found in only lengths 10, 11, 12, 14, 16, 17, 22, 25, and 26 (**Figure 5D**). Additionally, neither significant usage frequency difference of the *V* gene nor the *J* gene was observed between the groups (**Figure 5E**).

## Discussion

Herein, we present results of gene expression, BCR and TCR repertoire profiling, from 36,849 immune cells in patients with AD and NC at a single cell level for the fiing time. We revealed five major immune cell subsets, CD4+ T cells, CD8+ T cells, B cells, NK cells, and monocyte-macrophage cells, and displayed the proportion of cell subsets and gene expression patterns were distinct in AD from NC. Thirty-one cell type-specific key genes, including abundant *HLA* genes, and multiple immune-related pathways were identified in the present study. Moreover, we provided evidence for the changes in the TCR and BCR repertoire in AD. We also found that the proportion of high-frequency clonotypes of the TCR and BCR was increased and that the diversity of the TCR was decreased in AD correspondingly.

Compared with NC, various changes in the composition and functional state of immune cell subsets were presented in AD. The proportion of CD4+ T cells was significantly higher and that of CD8+ T cells was slightly lower in the AD group than in NC. Previous flow cytometry studies have shown similar results to the changes in the proportion of CD4+ T cells, but the change in the proportion of CD8+ T cells was contrary to this study (12–14). The features of distribution in T cells indicated that peripheral T cell subsets may have the disorder and adaptive immune dysfunction in patients with AD. CD4+ T cells and CD8+ T cells directed to Aβ or other antigens may promote the function of immune cells within the brain. Previous studies have shown that the elevated levels of activated CD4+ T cells and CD8+ T cells in peripheral blood is closely related to cognitive defects and MRI changes of specific brain regions in AD patients (11). Additionally, close communication exists between the peripheral immune response and the CNS.T cells can enter the brain and participate in AD pathogenesis. A single-cell sequencing study demonstrated that CD8+T cells with the same clonotype were detected both in blood and CSF, suggesting that peripheral CD8+ T cells appear to play a role after entering the brain (8). We speculate that the dysfunction in peripheral immune cell subsets may lead to the deterioration of the immune environment in the CNS. Moreover, most of the clusters were not overlapped between the AD group and NC, suggesting the abnormal gene transcription state in AD. Function and enrichment analysis for CD4+ T cells, CD8+ T cells, B cells, NK cells, and monocyte–macrophage cells revealed immune-related pathways in numerous aspects. Common pathways exist in different immune cell clusters, including “myeloid leukocyte activation”, “lymphocyte activation”, “adaptive immune system”, “cytokine production”, “immune response regulation signal pathway”, “leukocyte chemotaxis”, “hemostasis”, and “natural killer cell-mediated cytotoxicity”. This may imply a synergistic effect among different immune cells in the peripheral blood of patients with AD.

Thirty-one key genes were identified through the PPI network and module analysis. Module analysis is mainly used to cluster and construct functional modules in the huge gene (protein) network. In this study, module analysis is helpful to screen genes on the basis of the top 40 key genes obtained and exclude genes that are not closely related to the function of other genes. Additionally, in the module, combined with the cell type of each gene, it is easier to determine the biological function of the key gene. MCODE 1 comprises abundant HLA genes, mainly from B cells. The MHC encoded by *HLA* genes directly participated in the immune response. In the Alzheimer’s Disease Neuroimaging Initiative cohort study, it was found that the *HLA-DRB1/DQB1* allele variant may regulate the volume of the left posterior cingulate gyrus and affect the susceptibility of AD (15). Single-nucleotide polymorphism (rs1140317) in *HLA-DQB1* was significantly related to the thickness of the olfactory cortex and amyloid protein deposition in CSF (16). The differential expression of *HLA II* genes may imply different levels of immune response disturbance in AD. Other key genes also have a potential connection with AD. *ACTB* genes have been identified as important risk genes in proteomics study for CSF and in the plasma of patients with AD (17). The activated kinase LYN and SYK promote the development of local neuroinflammation in the brain of patients with AD, thus aggravating the severity of neurodegeneration (18). *GRB2*, *PPP1CA*, and *HSPA5* genes have been shown to affect tau phosphorylation and neurodegeneration (19–21). *UBB* is a member of the ubiquitin promoter gene family, and ubiquitin-proteasome activity is downregulated in the brain of patients with AD (22). *ITGA4* and *ITGB1*, two integrin family genes, were identified in the current study. The expression level of *ITGB1* was changed in PBMCs and the hippocampus (23), and the rs1143676 polymorphism of *ITGA4* increases the risk of AD (24). The *RHOA* gene encodes a small RhoGTP enzyme. The RhoA protein level decreased in the hippocampus of patients with AD, and it affects synaptic plasticity by regulating cytoskeleton dynamics (25). The *PDIA3* gene encodes an endoplasmic reticulum-associated protein, which regulates the protein folding process. A proteomic study showed that the PDIA3 protein is involved in Aβ-stimulated microglial activation (26). The *ALDH2* gene encoding glyoxylate dehydrogenase 2 is involved in the biological process of maintaining the mitochondrial function, and memory impairment occurred in *ALDH2* knockout mice (27). Except for these aforementioned genes, *CD247*, *CD3D*, *CD3G*, *PTPN6*, *ZAP70*, *CALR*, *IL7R*, *YWHAQ*, *EZR*, *MSN*, *PGD*, and TKT have not been reported to be associated with AD. Further work involving more in-depth experiments is needed to investigate their function in AD.

In the TCR and BCR repertoire analysis, we found amplification of high-frequency clonotypes in AD, and the proportion of high-frequency clonotypes for T and B cells was higher in the AD group than in NC. This finding may suggest that adaptive immune cells stimulated by specific antigens lead to clone expansion. A large number of autoantibodies exist in the peripheral blood of patients with AD, which distinguishes those with AD from healthy controls (28). Large amounts of autoantibodies were detected in the serum of patients with AD. These autoantibodies target autoantigens, including neurotransmitters and related receptors, such as the N-methyl-D-aspartate receptor, oxidized low-density lipoprotein, receptor for advanced glycation end products, and cellular adenosine triphosphate synthetase (29–31). The autoimmune response mediated by the B cells may serve as a damaging attack in response against normal tissues or as protective removal of waste fragments from tissue metabolites. In addition, based on the potential role of bacteria and viruses in AD pathogenesis, the specific clone amplification may be caused by bacterial and viral infections (32–34). Further study is needed to verify the epitopes matched with the high-frequency clonotypes.

We also detected some public clonotypes in both B and T cells of NC, but not in the AD group. This finding may be due to individual differences in the peripheral immune response of AD. Although the high-frequency clonotypes increased in the AD group, different patients with AD may respond to the stimulation of various antigenic determinants of the same or different antigens; therefore, it is difficult to obtain the public clonotypes with this small sample size.

V(D)J rearrangement in the transcription of T and B cells is accompanied by random insertion or deletion of nucleotides between V(D)J fragments, which determine the high variability of the CDR3 region and great differences in the amino acid sequence length. Our study revealed a significant difference in lengths 10, 11, 12, 14, 16, 17, 22, 25, and 26 in the BCR and in lengths 6, 10, 14, 15, 17, and 19 in the TCR between the AD group and NC. It has been noted that the structure of long-chain immunoglobulin H CDR3 is related to the autoimmune response (35, 36), and the distribution of the amino acid sequence length of the CDR3 region in memory T cells differed from that in naive T cells (37). The relationship between the CDR3 amino acid sequence length and function remains unclear. Moreover, skewed usage phenomenon of the TCR *V* gene and *J* gene with decreased diversity existed in AD. We speculated that this phenomenon suggests that when specific antigens stimulated T cells in the peripheral blood of patients with AD, free random rearrangement of the TCR *V* and *J* genes would be converted to antigen-dependent selective rearrangement. This would lead to specific clone amplification that would affect the abundance of clonotype repertoire and destroy the TCR diversity. The TCR diversity contributes to maintaining the stability of the immune system. The more abundant the clonotype repertoire, the more effective it is to resist various pathogens. On the contrary, the disruption of clonotype diversity may lead to more severe infections (38, 39).

Our study had some inherent limitations. First, we recognize the limitations for exploring AD public clonotypes posed by the relatively small sample size. Second, although we have identified the immune changes in peripheral blood of AD, the antigens leading to the clonal changes of T and B cells are not clear, and we did not perform analysis of epitopes matching the high-frequency clones and validation experiment. Third, due to the lack of CSF or brain sample, we cannot complete clone overlaps analysis between blood and CSF or brain. Fifth, although we have identified some key genes in AD, their functions weren’t be verified by *in vitro* or *in vivo* experiments. This content will be covered in our future research.

In conclusion, from an immunological perspective, the characteristics of PBMCs in AD were analyzed at the single-cell level in the present study. Abnormal changes of the immune cell composition and transcription state were detected in AD, which suggested disturbance of immune infiltration in the peripheral immune environment in AD. We also found AD-specific clonotype enrichment in the TCR and BCR, highlighting the potential role of the peripheral adaptive immune system in the pathogenesis of AD. These observations will facilitate a better understanding of the cellular basis of peripheral immune cells in AD and contribute to the development of immune-related biomarkers or therapy in future research.

## Data Availability Statement

The original contributions presented in the study are publicly available. This data can be found in the GEO database, accession number: GSE181279.

## Ethics Statement

The studies involving human participants were reviewed and approved by Medical Research Ethics Committee at Xuanwu Hospital. The patients/participants provided their written informed consent to participate in this study.

## Author Contributions

JJ and HX contributed to the conception and design of the research work. HX contributed to the data acquisition, data analyses, and generated figures. All authors are responsible for the drafting and revision of the article. All authors contributed to the article and approved the submitted version.

## Funding

This study was supported by the Key Project of the National Natural Science Foundation of China (81530036); the National Key Scientific Instrument and Equipment Development Project (31627803); the Key Project of the National Natural Science Foundation of China (U20A20354); Beijing Scholars Program; Beijing Brain Initiative from Beijing Municipal Science & Technology Commission (Z201100005520016, Z201100005520017).

## Conflict of Interest

The authors declare that the research was conducted in the absence of any commercial or financial relationships that could be construed as a potential conflict of interest.
