# Supplementary material for: Single-Cell RNA Sequencing of Peripheral Blood Reveals Immune Cell Signatures in Alzheimer’s Disease
Source: Front Immunol. 2021 Aug 9;12:645666. doi: 10.3389/fimmu.2021.645666 (PMC8382575; doi:10.3389/fimmu.2021.645666)
Supplement: Supplementary file 4 [file DataSheet_4.docx]

**Supplementary Figures and Tables**

Single-cell RNA-seq of peripheral blood reveals immune cell signatures in Alzheimer's disease

Hui Xu^1^, Jianping Jia ^1,2,3,4,*^

1.Innovation Center for Neurological Disorders and Department of Neurology, Xuanwu Hospital, Capital Medical University, National Clinical Research Center for Geriatric Diseases;

2.Beijing Key Laboratory of Geriatric Cognitive Disorders;

3.Clinical Center for Neurodegenerative Disease and Memory Impairment, Capital Medical University;

4. Center of Alzheimer's Disease, Beijing Institute of Brain Disorders, Collaborative Innovation Center for Brain Disorders, Capital Medical University

**∗Correspondence to:** Jianping Jia, Innovation Center for Neurological Disorders,

Department of Neurology, Xuanwu Hospital, Capital Medical University. 45

Changchun Street, Xicheng District, Beijing, China, 100053. Tel.: +86-10-

83199449. E-mail: jiajp@vip.126.com

**Method**

The inclusion criteria for patients with Alzheimer’s disease (AD) were as follows: 1) meeting the revised diagnostic criteria of the National Institute on Aging and the Alzheimer’s Association (NIA-AA) for probable AD and amyloid PET showed positive (1); 2) Mini-mental state examination (MMSE) <26 points and a global score of the clinical dementia rating scale (CDR) is 1 or 2; 3) no family history of dementia; 4) no infection-related symptoms in the past 3 months before blood collection; 5) no hormone supplements or immunosuppressant agent has been administered or applied externally; 6) no vaccination against influenza.

The exclusion criteria for patients with AD were as follows: 1) other nervous system diseases that may cause brain dysfunction (such as, Parkinson's disease, multiple sclerosis, brain tumour, brain trauma, depression metabolic encephalopathy, normal intracranial pressure hydrocephalus, encephalitis, epilepsy, etc.); 2) other systemic diseases that may cause cognitive decline, such as liver dysfunction, renal insufficiency, folic acid and vitamin B12 deficiency, thyroid dysfunction, severe anaemia, special infections (such as syphilis, HIV), alcohol and drug abuse, etc; 3) mental and nerve development retardation; 4) systemic diseases with obvious symptoms, such as heart failure, severe liver damage, tumour, drug dependence, drug addiction, etc; 5) other diseases related to immune system exist, such as lupus erythematosus, rheumatic arthritis, ankylosing spondylitis, etc.

The inclusion criteria for cognitively normal elderly were as follows: 1) MMSE ≥26 points and a global score of the CDR=0; 2) no family history of dementia; 3) no infection related symptoms within 3 months before blood collection; 4) No hormone or immunosuppressant has been administered or applied externally; 5) no vaccination against influenza.

The exclusion criteria for normal control patients were as follows: 1) The presence of any cerebral infarction, traumatic brain injury visualized on MRI; 2) a history of mental illness or congenital mental retardation; 3) Depression, schizophrenia and other mental system diseases. 4) systemic diseases with obvious symptoms, such as heart failure, severe liver damage, tumour, drug dependence, drug addiction, etc.; 5) other diseases related to immune system exist, such as lupus erythematosus, rheumatic arthritis, ankylosing spondylitis, etc.

**Reference**

1. McKhann GM, Knopman DS, Chertkow H, Hyman BT, Jack CR Jr, Kawas CH, et al. The diagnosis of dementia due to Alzheimer's disease: recommendations from the National Institute on Aging-Alzheimer's Association workgroups on diagnostic guidelines for Alzheimer's disease. *Alzheimers Dement* (2011) 7:263-9. doi: 10.1016/j.jalz.2011.03.005.

| **ID** | **diagnosis** | **sex** | **age** | **APOE** | **CDR global** | **CDR SOB** | **MMSE** | **MoCa** |
| --- | --- | --- | --- | --- | --- | --- | --- | --- |
| AD1 | AD | male | 77 | *APOE* ε2/4 | 1 | 4 | 24 | 18 |
| AD2 | AD | female | 66 | *APOE* ε3/3 | 1 | 7 | 12 | 5 |
| AD3 | AD | male | 60 | *APOE* ε2/4 | 2 | 9 | 5 | 4 |
| NC1 | NC | female | 77 | *APOE* ε3/4 | 0 | 0 | 30 | 27 |
| NC2 | NC | male | 65 | *APOE* ε3/3 | 0 | 0 | 30 | 27 |

**Supplementary Table S1:** Demographic and clinical information from Alzheimer’s disease (AD) and normal controls (NC).

| **Parameter** | **AD** | **NC** |
| --- | --- | --- |
| Estimated Number of Cells | 23,361 | 13,744 |
| Fraction Reads in Cells | 96.60% | 94.50% |
| Mean Reads per Cell | 71,439 | 82,389 |
| Median Genes per Cell | 1,508 | 1,191 |
| Total Genes Detected | 20,986 | 19,452 |
| Median UMI Counts per Cell | 4,281 | 3,232 |
| Number of Reads | 1,668,876,643 | 1,132,355,273 |
| Valid Barcodes | 85.30% | 66.10% |
| Valid UMIs | 99.90% | 100.00% |
| Sequencing Saturation | 88.70% | 83.80% |
| Q30 Bases in Barcode | 96.70% | 95.80% |
| Q30 Bases in RNA Read | 94.30% | 93.00% |
| Q30 Bases in RNA Read 2 | 91.90% | 91.20% |
| Q30 Bases in UMI | 96.20% | 95.30% |
| Reads Mapped to Genome | 94.20% | 94.40% |
| Reads Mapped Confidently to Genome | 82.80% | 68.50% |
| Reads Mapped Confidently to Intergenic Regions | 8.50% | 18.00% |
| Reads Mapped Confidently to Intronic Regions | 8.70% | 7.10% |
| Reads Mapped Confidently to Exonic Regions | 68.00% | 45.20% |
| Reads Mapped Confidently to Transcriptome | 58.30% | 29.40% |
| Reads Mapped Antisense to Gene | 7.60% | 14.50% |
| fraction of reads kept after aggregation | 91.60% | 98.20% |
| mean reads per cell after aggregation | 67115 | 78896 |

**Supplementary Table S2:** Overall quality of single cell RNA sequencing data of AD and NC groups.


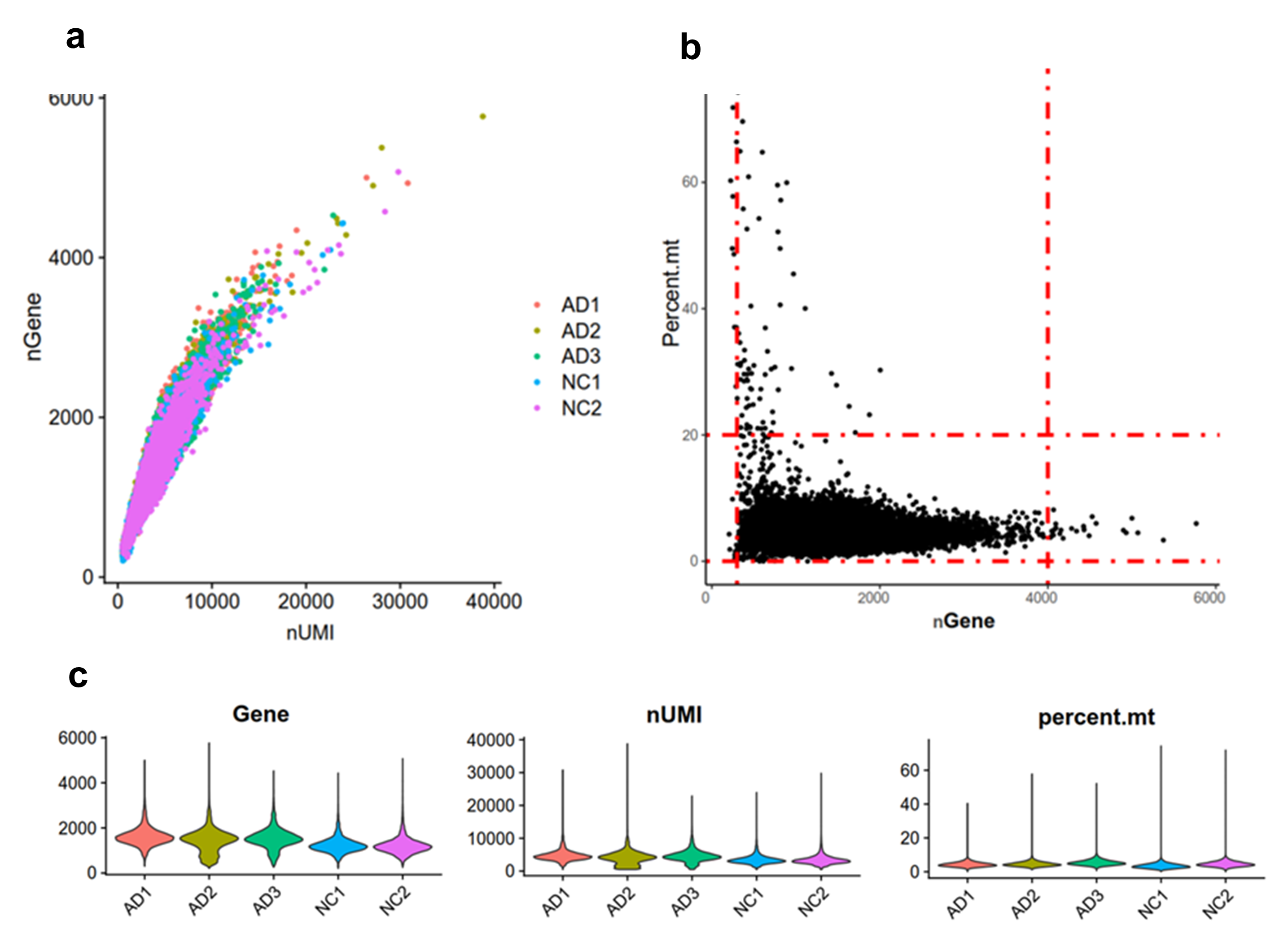


**Supplementary Figure S1** Quality of single-cell RNA-sequencing data in all sample.

a Scatter plot of correlation between detected gene and UMI number. The number of genes detected increased with the increase of cell UMI number. The correlation coefficient is 0.92. **b** Scatter plot of detected gene number and percentage of mitochondrion RNA, the cells in the red dotted line meets the quality control criteria.

**c** Violin plots of basic features of sequencing data, including number of gene, nUMI, and percentage of mitochondrion RNA. Thses data of each sample are comparable.


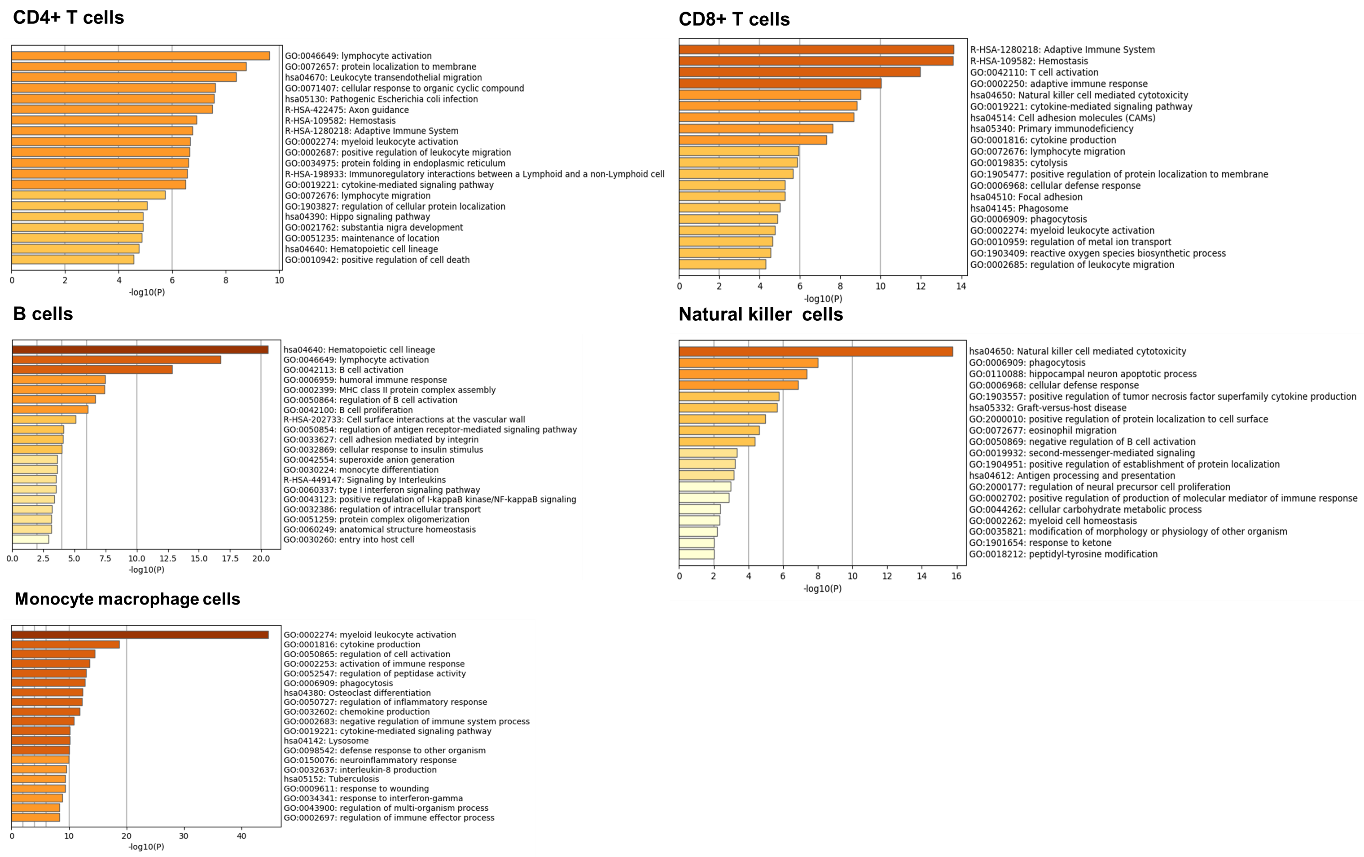


**Supplementary Figure S2** Functional and pathway enrichment analysis for CD4+ T cells, CD8+ T cells, B cells, natural killer (NK) cells, and monocyte macrophage cells subsets.

| **Name** | **GO** | **Category** | | **Description** | **LogP** | | **Log(q-value)** | | **Genes** | |
| --- | --- | --- | --- | --- | --- | --- | --- | --- | --- | --- |
| MCODE1 | R-HSA-202430 | | Reactome Gene Sets | Translocation of ZAP-70 to Immunological synapse | -35 | -30 | | CD3D\|CD3G\|CD247\|HLA-DPA1\|HLA-DPB1\|HLA-DQA1\|HLA-DQB1\|HLA-DRA\|HLA-DRB1\|HLA-DRB5\|ZAP70 | | |
| MCODE1 | R-HSA-389948 | | Reactome Gene Sets | PD-1 signaling | -33 | -29 | | CD3D\|CD3G\|CD247\|HLA-DPA1\|HLA-DPB1\|HLA-DQA1\|HLA-DQB1\|HLA-DRA\|HLA-DRB1\|HLA-DRB5\|PTPN6 | | |
| MCODE1 | R-HSA-202433 | | Reactome Gene Sets | Generation of second messenger molecules | -31 | -27 | | CD3D\|CD3G\|CD247\|HLA-DPA1\|HLA-DPB1\|HLA-DQA1\|HLA-DQB1\|HLA-DRA\|HLA-DRB1\|HLA-DRB5\|ZAP70 | |  |
| MCODE2 | hsa04510 | | KEGG Pathway | Focal adhesion | -9.9 | -7.8 | | ACTB\|RHOA\|GRB2\|ITGA4\|ITGB1\|PPP1CA | |  |
| MCODE2 | hsa04611 | | KEGG Pathway | Platelet activation | -8.8 | -6.9 | | ACTB\|RHOA\|ITGB1\|LYN\|PPP1CA | |  |
| MCODE2 | GO:0007159 | | GO Biological Processes | leukocyte cell-cell adhesion | -8.5 | -6.6 | | RHOA\|GRB2\|IL7R\|ITGA4\|ITGB1\|LYN | |  |
| MCODE3 | GO:0032388 | | GO Biological Processes | positive regulation of intracellular transport | -4.4 | -2.9 | | MSN\|SYK\|EZR | |  |
| MCODE3 | GO:1903362 | | GO Biological Processes | regulation of cellular protein catabolic process | -4.2 | -2.8 | | MSN\|UBB\|EZR | |  |
| MCODE3 | GO:0044257 | | GO Biological Processes | cellular protein catabolic process | -4.2 | -2.7 | | HSPA5\|MSN\|UBB\|EZR | |  |

**Supplemetary Table S3** Function and pathway enrichment analysis for key genes in three modules identified in protein-protein interaction networks by molecular complex detection (MCODE) algorithm.
